# Electrotherapy in the management of neuropathic corneal pain: narrative review

**DOI:** 10.3389/ebm.2026.10712

**Published:** 2026-02-06

**Authors:** A. V. Shanmathi, Mingyi Yu, Chang Liu, Isabelle Xin Yu Lee, Louis Tong, Yu-Chi Liu

**Affiliations:** 1 Trinity College Dublin, University of Dublin, Dublin, Ireland; 2 Regenerative Therapy Group, Singapore Eye Research Institute, Singapore, Singapore; 3 Ocular Surface Research Group, Singapore Eye Research Institute, Singapore, Singapore; 4 Department of Cornea and External Eye Disease, Singapore National Eye Centre, Singapore, Singapore; 5 Ophthalmology and Visual Sciences Academic Clinical Program, Duke-NUS Medical School, Singapore, Singapore

**Keywords:** corneal nerve dysfunction, hyperalgesia, nerve regeneration, neuropathic corneal pain, ocular pain management, transcutaneous electrical nerve stimulation

## Abstract

Neuropathic corneal pain (NCP) is a debilitating condition resulting from corneal nerve damage or dysfunction, leading to persistent ocular pain, discomfort and hypersensitivity. Conventional therapy with eye drops often provides inadequate relief, necessitating the need for alternative therapeutic approaches. This review explores the role of electrotherapy in managing NCP, including its mechanisms, clinical efficacy, and potential integration into multimodal treatment strategies. We examine current evidence on various electrotherapy modalities such as transcutaneous electrical nerve stimulation, neurostimulation, and microcurrent stimulation. These electrotherapies have the potential to modulate pain pathways, promote nerve regeneration, and restore corneal homeostasis. Emerging studies suggest electrotherapy may alleviate NCP by altering neural signaling and reducing hyperalgesia. Integrating electrotherapy into existing pain management strategies may enhance the outcomes for patients with refractory NCP. However, its clinical application remains limited by a lack of standardized protocols and robust clinical trials. Although electrotherapy presents a promising and non-invasive option for NCP management, further research is needed to optimize the treatment parameters and optimal duration, assess the long-term efficacy, and establish guidelines for clinical use.

## Impact statement

Neuropathic corneal pain (NCP) poses a significant clinical challenge with limited effective treatment options, affecting patients’ quality of life for its persistent ocular discomfort unresponsive to conventional therapies of dry eye. This comprehensive review advances the field by evaluating electrotherapy modalities, including transcutaneous electrical nerve stimulation, neurostimulation, and microcurrent stimulation as novel therapeutic approaches for NCP management. It provides critical new insights by synthesizing current evidence on the mechanisms of action of electrotherapy in promoting corneal nerve regeneration and modulating pain pathways, establishing a foundation for evidence-based clinical applications. This synthesis has a direct impact on clinical practice by identifying gaps in current treatment protocols and proposing standardized approaches for integrating electrotherapy into multimodal pain management strategies. This review aims to provide clinicians and researchers with necessary guidance for optimizing therapeutic outcomes, establishing a roadmap for future clinical trials and treatment protocol development, ultimately advancing the standard of care for patients suffering from this debilitating condition.

## Introduction

Neuropathic pain is defined by The International Association for the Study of Pain (IASP) as “pain caused by a lesion or disease of the somatosensory nervous system”. When it occurs in the cornea, it is referred to as neuropathic corneal pain (NCP). It causes patients to experience eye pain in the absence of any painful stimulus [1]. The cornea has a dense network of nerves in the body, making the cornea one of the most potent in pain generation in the body [2]. Small Aδ and C nerve fibers, which are part of the sensory and autonomic nervous systems, make up 70–90% of corneal nerves [2]. Coupled to the vital sensory function they hold, corneal nerves also sustain the functional integrity of the ocular surface by releasing trophic substances that support corneal homeostasis and by stimulating brainstem circuits that trigger reflex tear production and blinking [3].

## Clinical features and mechanisms of neuropathic corneal pain

### Etiology and origins of neuropathic corneal pain

NCP can be classified based on its underlying etiologies. Systemic causes of NCP include diabetes, systemic autoimmune diseases, medication-induced neuropathy (e.g., chemotherapy), and trigeminal neuralgia. Ocular causes include herpes simplex keratitis, dry eye disease (DED), refractive surgery, and ocular trauma [4]. In addition, NCP can be categorized based on the level of nervous system involvement, namely peripheral or central NCP [5]. Common causes of peripheral NCP include refractive surgery [6], chronic dry eye [7], and herpetic keratitis [8]. Peripheral sensitization occurs as a result of abnormal regeneration of corneal nociceptors and nerve fibers following corneal nerve injuries. Central sensitization results from elevated excitatory neurotransmitters due to chronic inflammation and, potentially, rewiring of pain perception pathways, which intensifies the perception of pain. Its common causes include small-fiber polyneuropathy, fibromyalgia, and radiation keratopathy [9].

### Associated comorbid conditions

NCP often coexists with conditions that affect pain perception and psychological well-being, such as depression, anxiety, fatigue, and sleep disturbances. These conditions are not causative factors but represent comorbidities that can influence the severity and impact of NCP. Moreover, NCP may be linked to comorbidities including anxiety, depression, and chronic pain syndrome such as migraine [10].

### Clinical presentations

Symptoms experienced by patients with NCP are described as dryness, burning sensation and hyperalgesia which are usually worsened by extreme temperatures, light (photoallodynia) and dry wind. Our group has identified that burning and light sensitivity are the two most common symptoms in NCP [11]. More focus is diverted to understanding the pathophysiology of pain which transitions from a protective physiological reflex to a more persistent and chronic condition [9].

Despite patients frequently reporting pain or pain-like symptoms, standard ophthalmological examinations often do not reveal objective or visible findings. This discrepancy can lead to delayed diagnosis and even many patients being misdiagnosed with dry eye disease (DED), which is a multifactorial condition characterized by an imbalance in tear film homeostasis and ocular symptoms, with tear film instability and hyperosmolarity, ocular surface inflammation and damage, and neurosensory abnormalities playing etiological roles [12]. Importantly, NCP and DED are not mutually exclusive and may coexist, or one may be a precursor or a sequel. These two conditions share partial overlap in symptomatology and underlying pathophysiological mechanisms. DED patients present with a range of symptoms including ocular dryness, burning, aching and tenderness, overlapping considerably with the symptom profile of NCP [7]. Additionally, neuroinflammation response is suggested to contribute to the pathogenesis of both conditions [7]. Due to the mismatch between the severity of symptoms and observable signs, patients’ symptoms are often dismissed as “psychological” or “functional,” leaving them feeling ignored and neglected, worsening their condition [10]. In terms of quality of life, Chin et al reported that patients with NCP had significantly worse quality of life on the majority of the items in the Ocular Pain Assessment Survey and Ocular Surface Disease Index questionnaires compared to those with DED, suggesting NCP is more debilitating than DED [10].

Although patients with NCP and DED share many common symptoms, the differentiating factors are imaging findings, proteomic patterns and clinical features which explain their distinct pathophysiologies. NCP is a neuropathic disorder with nerve dysfunction, corneal microneuromas, neuroinflammation, and pain that is disproportionate to clinical signs whereas DED is an inflammatory and tear film disorder accompanied with epithelial damage, immune activation, and symptoms that are correlating with objective findings. Microneuromas are the key differentiator and NCP patients exhibit higher numbers, larger area and larger perimeter of microneuromas than DED patients. NCP patients have elevated levels of tear proteome associated with neuronal activity and neuroinflammation whereas DED showed proteomic patterns dominated by inflammatory mediators [7]. Treatment wise, some patients with NCP do not respond to conventional treatments used to treat DED and thus differentiating them is essential to offer more effective treatment [13].

### *In vivo* confocal microscopy (IVCM) imaging findings of neuropathic corneal pain

**FIGURE 1:**
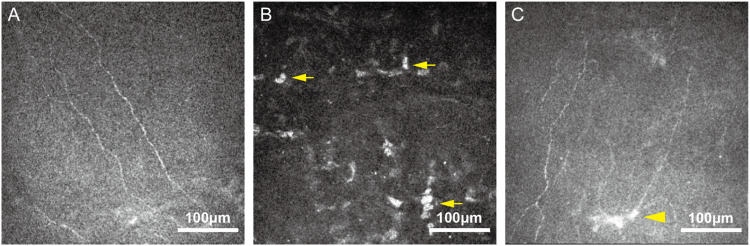
Representative *in vivo* confocal microscopy (IVCM) images from patients with NCP. **(A)** Decreased corneal nerve density and nerve fiber fractal dimension. **(B)** Activated keratocytes (arrows), appeared as patchy areas of increased stromal reflectivity. **(C)** Microneuromas (arrowhead) manifested as irregularly shaped enlargements of terminal nerve endings with poorly defined margins and variable hyper-reflectivity.

*In-vivo* confocal microscopy (IVCM) has been extensively used to visualize the corneal nerve plexus and corneal cells [14]. Through IVCM evaluation, decreased corneal nerve densities, decreased nerve fiber fractal dimension, increased corneal nerve fiber width, activated keratocytes, smaller corneal epithelial cell size, and an increased number, area and perimeter of micro neuromas in patients with NCP were identified (Figure 1) [11, 15]. Previous study suggested that the presence of microneuromas on IVCM could serve as a biomarker to distinguish NCP from DED, with high sensitivity and specificity [16]. However, recent evidence indicates that microneuromas can be observed not only in patients with NCP, but also in painless DED and even in healthy subjects [17]. From a diagnostic perspective, the mere presence of microneuromas therefore appears insufficient to indicate corneal neuropathic alterations. Quantitative microneuroma metrics, such as perimeter and area, may be required to improve diagnostic specificity [17].

### Pathogenesis and pathways involved in neuropathic corneal pain

NCP is characterized by increased release of excitatory neurotransmitters at presynaptic terminals, ectopic firing of nerve fibers, nerve sprouting and rewiring, spontaneous generation of action potentials, abnormalities in signal conduction, and transformation of non-nociceptive sensory fibers into nociceptive ones [18]. Nociceptors are important for pain perception and can produce action potentials in response to thermal, mechanical, chemical, or polymodal stimuli [19]. The nociceptors are centrally connected to higher-order somatosensory pain pathways and the thalamus, where pain is perceived. Tissue damage and inflammation of the ocular surface cause peripheral axonal injuries and release of pro-inflammatory mediators, which lower the activation thresholds of ion channels in affected nerve endings. These sensitizing effects may spread to adjacent nociceptors, enhancing peripheral nociceptive signaling and ultimately leading to peripheral sensitization [20].

In addition to the ascending pathways that transmit pain signals, descending modulatory pathways also play a critical role in NCP by contributing to central sensitization and heightened pain perception [21]. Descending inhibitory activity is believed to originate from the reticular system and thalamus, activating central gamma-aminobutyric acid (GABA) receptors, modulating interneurons, and ultimately altering ascending trigeminal pain signals. Normally, interneurons release inhibitory neurotransmitters that suppress nociceptive signaling [22]. However, persistent insult reduces the inhibitory effect of GABA on ascending pathways due to changes in chloride currents [23]. As a result, signals in the ascending pain pathways intensify, contributing to the perception of chronic pain [24]. Furthermore, descending modulation of the trigeminal nuclei can be either inhibitory or facilitatory. Inhibitory control predominates under physiological conditions, while a shift toward facilitatory modulation following tissue or nerve injury may contribute to the development of chronic pain [25].

Genetic polymorphisms can also affect corneal neurophysiology and inflammation by altering the expression of neurokines and neuromediators which influences the clinical presentation and severity of NCP. A recent report discovered a single nucleotide polymorphism (rs140293404), an intronic variant in the gene ENSG00000287251, that has met the genome-wide significance threshold for NCP [26]. Genetic polymorphisms may explain why some are more susceptible to NCP.

Continuous stimulation of corneal nerves causes the release of glutamate from presynaptic afferent neurons, which activates two types of glutamate receptors: N-methyl-D-aspartic acid receptors (NMDAR), responsive to strong stimuli, and α-amino-3-hydroxy-5-methyl-4-isoxazolepropionic acid receptors (AMPAR), sensitive to weaker signals [27]. The activation of NMDAR results in intense and prolonged neural responses [9]. Peripheral axonal injury induces the release of inflammatory mediators that decrease the threshold potentials of ion channels in nerve endings, intensifying neural responses. Moreover, mechanical stimuli in the cornea are transduced by Piezo2 channels, which are low-threshold mechanically activated channels expressed in trigeminal sensory neurons innervating the cornea [28]. Research indicates that Piezo2 contributes to various forms of mechanoreceptive sensitization, including mechanical allodynia induced by inflammatory responses or injury [29, 30]. In NCP, alterations in Piezo2 channel function may affect innate reflexes and cause mechanical hypersensitivity [31]. Recurrent dysregulation of Piezo2 signaling may facilitate nociceptive sensitization and influence long-term central nervous system responses even after the offending stimulus is removed [32]. A previous study using Piezo2 conditional knockout mice demonstrated that Piezo2 channels in corneal neurons are directly involved in corneal mechano-nociception [33]. However, systemic inhibition of Piezo2 for neuropathic pain relief is not feasible due to its essential role in mechanotransduction processes across multiple organs, including touch, proprioception, and interoception [33]. Despite this limitation, topical inhibition of Piezo2 in the cornea may represent a promising strategy to alleviate discomfort and pain associated with corneal mechanical irritation and warrants further investigation. Additionally, photophobia and photoallodynia are common symptoms in NCP. The presence of melanopsin expression in trigeminal neurons suggests the existence of a functional neural pathway that permits light to influence various sensory processes [34].

Neurotrophins released during neuroinflammation in NCP act as retrograde signaling molecules. Evidence from trigeminal ganglion studies demonstrate that neurotrophins can induce changes in ion channel expression and membrane excitability in sensory neurons. Although the specific role of neutrophils in NCP remains incompletely understood, findings extrapolated from peripheral nerve injury models indicates they may promote nerve depolarization and contribute to altered pain perception through modulation of neuronal properties [35]. Chronic neuropathic nerves may further lead to reorganization, resulting in more excitable and abnormal firing patterns [36]. Furthermore, recent research indicates that immune cells, including dendritic cells, release neuropeptides and neurokines that modulate neuronal excitability, contributing to peripheral sensitization [37]. These collectively exacerbate neuroinflammation, which perpetuates the cycle of NCP [38]. As time passes, sustained peripheral input may facilitate the development of central sensitization, where central neurons become increasingly responsive to similar levels of pain, resulting in heightened pain perception [39].

On a molecular level, neurokines like Interleukin- 1 Beta (IL-1β) and Tumour Necrosis Factor-Alpha (TNF-α) bind to receptors on corneal sensory afferents, modulating neuronal excitability and lowering thresholds for generation of action potential, thus, facilitating the release of neuropeptides such as substance P [37, 38]. Substance P directs immune cells to the terminals of nociceptors, where they release neurokines that contribute to neuropathic pain [40]. Substance P and Calcitonin Gene-Related Peptide (CGRP) have prolonged effects on corneal nociceptors and are strongly implicated in sensory hypersensitivity [35]. Neurokines and neurotrophic factors promote the reorganization and increased sprouting of peptidergic nerve fibers [41]. Additionally, nerve growth factor (NGF), a neurotrophin, increases in the tears of patients with NCP [11], altering the expression of transduction molecules in C- and Aδ-fibers, increasing their excitability [42, 43]. Our group further identified the significantly dysregulated tear proteins in NCP. Specifically, the levels of metallothionein-2, creatine kinase B-type, vesicle-associated membrane protein 2, neurofilament light polypeptide and myelin basic protein were significantly over-expressed [11].

## Current management of neuropathic corneal pain

Treatment of NCP involves a complex, long-term and multi-step approach. Lubricants lower tear osmolarity and dilute pro-inflammatory mediators, while topical steroids or cyclosporines have been a cornerstone in anti-inflammatory treatments due to their ability to inhibit the production of cytokines, prostaglandins, and leukotrienes. They also prevent leukocyte migration, contributing to their overall effectiveness in reducing inflammation [44]. Autologous serum tears (AST) have been shown to improve corneal nerve regeneration, relieve corneal pain and photoallodynia as they contain growth factors and anti-inflammatory components [45]. When symptoms of NCP are due to central sensitization, systemic pharmacotherapy such as tricyclic antidepressants, anticonvulsants, low-dose naltrexone, serotonin-norepinephrine inhibitors, sodium channel blockers and calcium channel ligands are used. They may also help in treating peripheral sensitization and speed up relief measures [46]. Other ocular treatments include cryopreserved amniotic membrane (PROKERA®, Bio-Tissue, Miami, FL) that confer anti-inflammatory and neurotropic effects and extended-wear soft bandage contact lenses or scleral lenses that protect the ocular surface and promote healing by reducing mechanical irritation [47]. Table 1 summarizes the current common management strategies for Neuropathic Corneal Pain and its mechanism of action.

**TABLE 1:** Current management strategies for neuropathic corneal pain.

Treatment	Mechanism of action
Lubricants	Lubricants dilute pro-inflammatory mediators and lower tear osmolarity [44]
Topical steroids	Topical steroids prevent leukocyte migration by inhibiting cytokine, prostaglandin, and leukotriene production [44]
Topical cyclosporine	Cyclosporine suppresses T-cell activation and reduces inflammatory cytokines [44]
Autologous serum tears (AST)	AST contains growth factors and anti-inflammatory components that promote corneal nerve regeneration and reduce pain and photoallodynia [45]
Cryopreserved amniotic membrane (PROKERA®)	Confers anti-inflammatory and neurotrophic effects [47]
Bandage contact lenses/scleral lenses	These lenses protect the ocular surface, reduce mechanical irritation, and promote healing [47]

In addition to the above-mentioned treatment, electrotherapy is an emerging option that utilises electrical stimulation (ES) to modulate pain and is being explored as a potential treatment for managing NCP via pain modulation, neuroplasticity and reduction of sensitization [48].

## Electrotherapy as a therapeutic approach for neuropathic pain

### Electrotherapy in peripheral neuropathic pain

Electrotherapy has been used in the treatment of peripheral neuropathic pain. It involves using electrical impulses to stimulate nerves in the affected area, helping in pain reduction and promoting healing [49]. The analgesic effects of electricity were attributed to the activation of the descending inhibitory pathway, increasing the production of endogenous opioids [50] and other neurochemical compounds such as serotonin [51], GABA [52], and adenosine [53]. The pain reduction could also be attributed to the gate-control theory proposed by Melzack and Wall, which states that pain is modulated by inhibiting small afferent nociceptive fibers through the activation of larger afferent fibers of the spinal cord [54]. This leads to the activation of inhibitory interneurons and reducing nociceptive signaling [55].

Among different electrotherapies, Transcutaneous Electrical Nerve Stimulation (TENS) is a widely used form that involves placing electrodes on the skin near the painful area that deliver low-frequency electrical impulses to stimulate the affected nerves. Previous studies suggest that TENS may reduce nociceptive signaling by decreasing nociceptor activity, modulating the expression of pain-related ion channels, thus inhibiting nociceptor neurotransmission [56]. β-endorphins and methionine-enkephalin levels, which interact with opioid receptors, increase after high-frequency TENS, which may contribute to reduced release of glutamate and substance P in the spinal cord [55].

In diabetic neuropathy, conventional treatments for painful diabetic neuropathy have essentially focused on drug therapies such as gabapentin, pregabalin, duloxetine, and tricyclic antidepressants. However, these drugs are associated with notable side effects involving dizziness, sedation, peripheral edema and anticholinergic effects including cardiac arrhythmias [9]. Despite these treatments, many patients experience persistent pain, incurring substantial indirect and direct economic costs [57]. ES is a potentially safer option due to its minimal contraindications and absence of known drug interactions [58]. Clinical studies reported that ES enhances peripheral cutaneous circulation, potentially through stimulation of sympathetic nerves [59]. Although improved cutaneous circulation has been associated with pain relief in neuropathic conditions [60, 61], the specific underlying mechanisms remain incompletely understood. Additionally, ES has been shown to increase vascular endothelial growth factor (VEGF) levels. VEGF improved the microcirculation associated with neuropathy, which may enhance nerve function [62]. Other studies have demonstrated notable increases in beta-endorphin levels [63], heightened expression of CGRP [64], reduction in inflammatory markers [65], and increased NGF after ES [66]. ES also diminishes pain by inhibiting nociception at the presynaptic level within the dorsal horn, thereby preventing the central transmission of pain signals [67]. Kumar et al reported that TENS resulted in a reduction in pain and discomfort in 83% of patients, although the symptoms recurred approximately after 1 month upon termination of TENS [68]. However, a systematic review presented only a slight reduction in pain intensity favoring TENS. The considerable variation in treatment protocols, such as the intensity, frequency, and duration of TENS application, made it challenging to synthesize the research findings effectively [69].

In this review, we aim to explore the role of electrotherapy in the management of NCP, focusing on its underlying mechanisms, efficacy, and clinical applications. We evaluate current evidence, highlight emerging technologies, and discuss future directions for integrating electrotherapy into clinical practice.

### Search strategy and selection criteria

**FIGURE 2:**
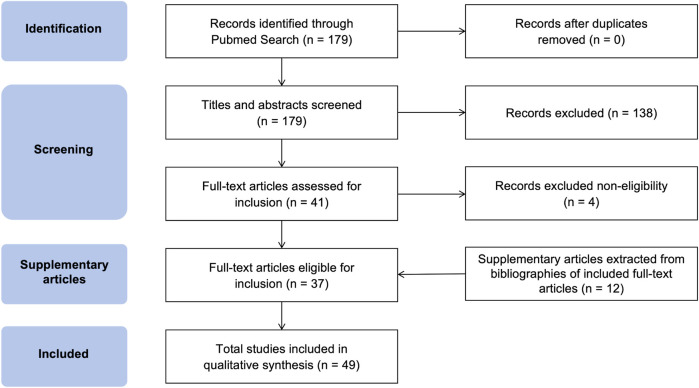
Flow diagram of the literature selection process for the present review.

An electronic literature search was conducted on the PubMed database to explore the effects of electrotherapy in the management of NCP. The search utilized a combination of the following terms: “(Electrotherapy OR Electrical Stimulation OR TENS OR Neuromodulation)” AND “(Neuropathic Corneal Pain OR Keratoneuralgia OR Corneal Neuropathy OR Corneal Pain OR Neuropathic Ocular Pain)” AND” (Management OR Treatment OR Intervention)”. Methods of stimulation such as transcranial direct current are not within the scope of this narrative review because it is unclear which pain pathways are involved. The search spanned from the database’s inception to December 18, 2024, with no filters applied. The initial search yielded 179 references published between 1955 and 2024. Articles of various study types, including clinical trials, meta-analyses, randomized controlled trials, reviews, and systematic reviews, relevant to the application of electrotherapy in NCP, were included after duplicate removal. Exclusion criteria included non-English articles and those without full-text availability. Following the application of inclusion and exclusion criteria and a screening of titles and abstracts for relevance, 45 references were deemed suitable for further review (Figure 2). Full-text versions of these articles were assessed for eligibility. Additional relevant studies identified through cross-referencing were incorporated to provide a broader discussion, encompassing the application of electrotherapy in neuropathic pain.

### Electrotherapy in neuropathic corneal pain

Recent advancements in neurostimulation technologies have led to improved outcomes in reducing chronic corneal pain, particularly in cases where conventional treatments have proven ineffective. Hence, electrotherapy has emerged as a treatment option for managing NCP, offering a potential alternative to traditional pharmacological treatments or as adjunct therapy together with conventional therapy. By delivering targeted ES to affected nerves, this therapy aims to modulate pain signals and potentially promote nerve regeneration [70].

#### Transcutaneous trigeminal ganglion stimulation

The pain signals from the cornea are transmitted via the trigeminal nerve, which connects with second-order neurons in the trigeminal subnucleus caudalis. These neurons send signals to the thalamus, where they link with third-order neurons that extend to the sensorimotor cortex and paralimbal region, both of which play a role in the emotional experience of pain [71]. Pain management may be improved by targeting different points along these pathways. A single case report described the use of transcutaneous trigeminal ganglion stimulation in a patient who developed severe NCP following laser-assisted *in situ* keratomileusis (LASIK). An electrode array was implanted into the trigeminal ganglion through the left foramen ovale, in which an electrode was inserted and positioned toward the V1 branch of the trigeminal ganglion. Intraoperative testing with the patient awakes confirmed paresthesia in the V1 region without causing corneal anesthesia. Despite unilateral stimulation, the patient reported immediate relief from bilateral pain. Symptom relief was maintained for 8 months but the pain re-emerged when the electrode leads migrated [72]. While these observations provide preliminary evidence suggesting that trigeminal ganglion stimulation may have effectiveness on the treatment of NCP, the evidence limited to a single case. Further studies are required to establish the safety, efficacy, and clinical applicability of this approach. Additionally, as an anecdotal observation, it lacks a control group to compare the outcomes of the neuromodulatory treatments with other treatment modalities or a placebo. Without such a comparison, it is challenging to ascertain whether the observed outcomes were directly due to the treatments or influenced by other factors, such as the natural variation of symptoms over time or psychological elements affecting the perception of pain. The report mentions complications such as the migration of the electrode and catheter, which required additional procedures and revisions. These complications suggest that whilst the treatments may provide short-term benefits, they may also carry risks of failure or require further interventions. It is also important to note that the effectiveness of these treatments may rely on the expertise of the healthcare team and the availability of specific equipment, which could limit the broader applicability. Moreover, the durability of symptom relief and the long-term safety profile remain uncertain, which warrants long-term follow-up studies. Consequently, the clinical applicability of trigeminal ganglion stimulation for NCP remains limited, and further well-designed, controlled studies are required before its therapeutic role can be defined.

#### Transcutaneous electrical stimulation

The pain modulation effects of electrotherapy in NCP may result from its nerve-stimulating effects. A study focused on determining the effect of transcutaneous electrical stimulation (TES) on corneal nerve regeneration in rabbits that underwent superficial lamellar keratectomy (SLK) [73]. TES was administered for 28 days following the corneal nerve injury secondary to SLK. Corneal sensitivity was measured, and changes in the corneal tissue were observed through Western blotting, real-time polymerase chain reaction (PCR), and immunofluorescence in which proteins involved in corneal nerve regeneration and wound healing were evaluated. Compared to post-treatment day 1, corneal sensitivity increased on day 7 in both the control group and the 2-Hz and 20-Hz treatment groups. Notably, the corneal sensitivity in the 2-Hz and 20-Hz groups (both 1.5 ± 0.0 cm) was significantly higher than that in the control group (0.8 ± 0.3 cm) on day 7. Western blotting showed that the expression of SPRR1A, a regeneration-associated protein, was significantly increased in the 2-Hz group on days 1 and 7 compared to the control and 20-Hz groups. PCR results showed a significant increase in NGF on day 1 in the 2-Hz group compared to the other groups. Furthermore, immunofluorescence demonstrated notable nerve regeneration, with a significantly higher density of subbasal nerve plexus and nerve terminal in the 2-Hz group. These results collectively suggest that TES promoted corneal nerve regeneration and enhanced corneal sensitivity in the SLK rabbit model. However, this study only tested only two frequencies (2 Hz and 20 Hz) of ES. Although corneal nerve regeneration was assessed, the authors did not directly measure pain or other subjective symptoms.

In another randomized clinical study, TES improved corneal nerve sensitivity 3 months after LASIK, potentially by promoting accelerated regeneration of corneal nerves [74]. Among the total of 40 eyes from 20 patients, one eye was randomly assigned to receive 60 min of TES at 20 Hz, while the other eye served as a control. Corneal sensitivity was assessed using the Cochet-Bonnet esthesiometer at four peripheral and five central locations within the LASIK flap, both preoperatively and at multiple postoperative intervals (1 day, 1 week, 1 month, and 3 months). Notably, this study focused on postoperative corneal nerve sensitivity and regeneration and did not include direct assessments of pain severity or TES-induced pain relief.

It has been proposed that ES promotes nerve and tissue regeneration via an increased calcium influx into neurons. When ES was applied to cultured dorsal root ganglion neurons, it significantly enhanced neurite outgrowth [75]. Similar to peripheral nerve injuries, corneal nerves also rely on calcium-dependent processes for regeneration. Blocking calcium waves—whether by inhibiting voltage-gated calcium channels or calcium release from intracellular stores like the endoplasmic reticulum—has been shown to impair nerve regeneration. ES is hypothesized to increase retrograde calcium signals, thereby activating nerve repair [76]. ES might also decrease the time needed for corneal nerve fibers to reconnect to their target areas, potentially reducing the chronic pain associated with delayed regeneration in NCP [75]. While the functional recovery of corneal nerves due to ES has been demonstrated, direct evidence pertaining to structural recovery and the physiological mechanisms involved remains ambiguous and requires further research.

#### Transcutaneous electrical nerve stimulation

More recently, Zayan et al presented the use of TENS for managing chronic ocular pain. Fourteen patients with chronic ocular pain who had previously received treatments such as topical medications and oral analgesics that provided insufficient relief were recruited [77]. These patients received TENS treatment for a minimum of 3 months, with a median duration of use being 6.5 months. All patients were able to integrate TENS into their ocular pain management regimen. The parameters such as pulse width, amplitude, and frequency were adjusted to each patient’s comfort level. Patients were instructed to use the TENS device up to three times daily, adjusting the intensity to their comfort. The device operated at pre-set frequencies of 5000/5100 Hz, creating a 100 Hz beat frequency. Four electrodes were placed near the trigeminal nerve branches on the forehead and temple. Patients adjusted the intensity by increasing the signal until they no longer felt pain, then lowering it slightly. This process was repeated on the right side first, followed by the left. Patients initially reported a median device usage of 14.0 times per week (range: 3 – 21). However, their usage decreased to a median of 3.0 times per week as they began experiencing the benefits by the time of the last follow-up. The findings suggested that TENS therapy resulted in significant, long-lasting pain reduction in most of the study patients, where 90% patients indicated a subjective reduction in pain. Pain intensity was reduced by about 27.4%. None of the patients reported experiencing any adverse effects. This study provides preliminary evidence for TENS as a treatment for NCP, with the potential to complement pharmacological therapies. It opens avenues for further research into the long-term efficacy of TENS in NCP. However, concerning the treatment protocol, patients used the TENS device at varying frequencies. Further studies on the optimal frequency, standardized protocol and long-term effectiveness of TENS are warranted for wider clinical applications. Moreover, participants were taking multiple systemic analgesics, which is a significant confounding factor that limits the ability to attribute the analgesic effects solely to TENS. Future studies with better control for the use of systemic analgesics may isolate the effects of TENS more clearly.

It is important to recognize that sympathetic efferent hyperactivity can often play a significant role in the development and persistence of neuropathic conditions, including NCP. TENS has been found to influence sympathetic activity through a sympatholytic effect, which may help explain its pain-relieving effects [78]. Unlike oral medications such as opioids and Nonsteroidal Anti-inflammatory Drugs that often present with systemic side effects, TENS may have fewer systemic side effects due to localized application. This is especially important for older patients with other comorbid conditions where one should be more cautious with the side effect profile of certain drugs.

#### Intranasal neurostimulation (ITNS)

Recently, ITNS has emerged as a novel electrotherapeutic approach targeting the neurophysiology of the lacrimal functional unit and has recently gained attention in the management of DED and ocular pain. TrueTear (Allergan, San Diego, CA), an ITNS device approved for the treatment of DED, delivers adjustable electrical pulses (up to 13 V or 5 mA at 30–60 Hz) to stimulate the anterior ethmoidal nerve, thereby activating the nasolacrimal reflex [70]. Clinical studies have demonstrated a favorable safety profile, with mild, self-limited nasal discomfort and occasional epistaxis being the most commonly reported adverse events [79]. Both animal and human studies further suggest that intranasal stimulation not only increases tear secretion but also promotes lipid and mucin release, supporting a more comprehensive restoration of tear film components [80, 81]. An interventional case series involving DED patients with ocular pain reported that ITNS significantly increased tear volume and reduced symptom severity, including dryness and ocular pain [82]. Notably, improvements in pain and dryness were not correlated with changes in tear volume, indicating that analgesic effects of ITNS may occur independently of tear production. ITNS is hypothesized to share mechanisms with TENS [83]. Stimulation of large-diameter Aβ fibers in the anterior ethmoidal nerve may presynaptically inhibit nociceptive input from small corneal C fibers at the level of the spinal trigeminal nucleus, thereby reducing pain perception in the somatosensory cortex. However, the study did not assess corneal nerve morphology, which may be relevant to the outcome of NCP. Additionally, only a single ITNS session was performed, and the durability and reproducibility of the treatment effects after multiple sessions require further investigation.

Another study enrolled patients with peripheral or mixed NCP and evaluated the effects of daily ITNS over a 90-day period [84]. The results indicated that ITNS could effectively alleviate pain symptoms, with a more pronounced reduction observed in patients reporting burning sensations. However, heterogeneity in treatment response was noted, as some patients appeared to develop tolerance to repeated treatment over time. Taken together, ITNS may present a promising adjunct treatment for NCP and future prospective randomized trials are warranted.

#### Extranasal neurostimulation (EXNS)

EXNS is a targeted electrotherapy approach that stimulates the external nasal nerve, a terminal branch of the anterior ethmoidal nerve, which is an extraconal branch of the nasociliary nerve [85]. Although the external nasal nerve is initially regarded as a sensory nerve containing Ab fibers, it has been shown to stimulate tear production by activating the lacrimal functional unit [85]. According to the gate control theory, stimulation of these Aβ fibers via EXNS may inhibit pain signals transmitted by smaller-caliber Aδ and C fibers originating from the cornea [86]. This inhibition reduces the transmission of pain signals to second-order neurons in the trigeminal nucleus, ultimately decreasing the pain signals reaching the somatosensory cortex [83]. A pilot study investigating the efficacy of EXNS in patients with refractory peripheral or mixed NCP reported that following a single session of EXNS, patients had an average pain reduction of 54.88% as measured by the Visual Analog Scale (VAS) [87]. Subgroup analysis showed a 68.40% decrease in pain intensity for patients with peripheral NCP and a 43.61% reduction for those with mixed NCP. Moreover, 63.63% of NCP patients experienced at least a 50% improvement in pain, 9.09% had a 30–49.9% improvement, while 27.27% showed less than 30% improvement. However, there existed significant variation in the responses of patients in this study. These findings suggest that EXNS may serve as an adjuvant therapy to alleviate pain in NCP patients, especially those with a peripheral pain component. However, this study primarily assessed pain outcomes and did not evaluate changes in tear film parameters. Future research is needed to explore the long-term efficacy of EXNS, its impact on tear film quality, and whether it can enhance the effects of other treatments in managing NCP.

#### Scrambler therapy

Scrambler therapy is another non-invasive electrotherapeutic approach designed to remodulate aberrant pain signaling and has emerged as an option for the management of various forms of neuropathic pain, and its efficacy has been supported by multiple randomized and non-randomized clinical trials [88]. Scrambler therapy delivers constantly changing electrical signals across the affected dermatome(s) to substitute pathological “pain” information with synthetic “non-pain” signals. These signals engage peripheral nerve fiber endings, generate action potentials, and transmit altered sensory information to the spinal cord and brain [89]. This process is thought to modulate central pain processing, potentially through redistribution of cerebral blood flow from pain-related regions toward frontal inhibitory centers [89]. In addition, successful therapy has been associated with normalization of serum neuroinflammatory mediators, including NGF, suggesting a broader neuromodulatory and anti-inflammatory effect [90]. A typical treatment session lasts 30–45 min and may be repeated up to ten times or until adequate pain relief is achieved. In cases of pain recurrence, booster sessions (usually two to three treatments) can be administered and often result in a more sustained remission [88]. A case series involving 3 patients with unilateral NCP who were refractory to conventional topical and systemic treatments or experienced intolerable side effects demonstrated meaningful functional improvement and a reduced reliance on systemic analgesics, although complete pain resolution was not achieved [91]. These findings suggest that Scrambler therapy may be a potential treatment approach for NCP. All patients had pain associated with a documented extra-ocular trigeminal nerve injury, limiting the generalizability of these findings. Consequently, the effectiveness of Scrambler therapy in bilateral NCP or in cases arising directly from ocular surface disease remains uncertain and warrants further investigation. While Scrambler therapy may offer more durable pain modulation compared with conventional transcutaneous electrical nerve stimulation, its clinical use is constrained by the need for specialized equipment and trained providers, limiting accessibility. Table 2 summarizes the key findings of publications on the application of electrotherapy on NCP.

**TABLE 2:** The application of electrotherapy in the management of neuropathic corneal pain.

Study	Study type	Electrotherapy	Key findings	Conclusion
Sayegh et al. [72]	Case study	Percutaneous stimulation of the trigeminal ganglion in a 32-year-old woman with severe corneal neuropathic pain after LASIK	Complete symptom relief after electrode implantation, but pain recurred with lead migration	Trigeminal ganglion stimulation is effective for treating severe NCP in LASIK patients unresponsive to conventional therapies
Yoo et al. [73]	Experimental study	TES at 2-Hz & 20-Hz frequencies for 28 days on New Zealand white rabbits with corneal nerve damage induced by SLK	Increased corneal sensitivity in both TES groups with significant increase in SPRR1a, NGF and nerve regeneration observed in the 2-Hz group	TES promotes corneal nerve regeneration in the rabbit SLK model, with 2-Hz frequency being more effective than 20-Hz frequency, indicating potential for clinical applications in corneal nerve degeneration
Zayan et al. [77]	Retrospective study	Home use of TENS device in ten patients with chronic ocular pain unresponsive to conventional treatments	Overall pain intensity decreased by 27.4% post-treatment with no adverse events reported	Integration of TENS into the long-term management of ocular pain leads to improvements in overall pain intensity
Farhangi et al. [82]	Retrospective case series study	A single session of ITNS treatment in DED patients with ocular pain	ITNS significantly increased tear volume and reduced the severity of dryness and ocular pain symptom. Improvements in pain and dryness symptoms not correlated with in tear volume changes	ITNS may present a promising adjunct treatment for NCP and the analgesic effects of ITNS may occur independently of tear production
Olcucu et al. [84]	Prospective study	Daily ITNS treatment in patients with peripheral or mixed NCP over a 90-day period	ITNS reduced pain scores evaluated by ocular pain assessment survey, with a more pronounced reduction observed in patients reporting burning sensations	ITNS can be effective in relieving pain symptoms in most patients with peripheral and mixed NCP, in particular in patients with burning
Koseoglu et al. [87]	Retrospective pilot study	A single session of EXNS in patients with refractory peripheral or mixed NCP	NCP patients had an average pain reduction of 54.88%. Peripheral NCP patients reported a 68.40% pain reduction, while mixed NCP patients reported a 43.61% pain reduction	EXNS may serve as an adjuvant therapy to alleviate pain in NCP patients, especially those with a peripheral pain component
Karakus et al. [91]	Case series study	Scrambler therapy in 3 patients with unilateral NCP who were refractory to conventional treatments or experienced intolerable side effect	All patients had pian relief and a reduced reliance on systemic analgesics, although complete pain resolution was not achieved	Scrambler therapy may be a potential treatment approach for NCP. Its effectiveness in bilateral NCP or in cases arising directly from ocular surface disease warrants further investigation

LASIK, laser-assisted *in situ* keratomileusis; NCP, neuropathic corneal pain; TES, transcutaneous electrical stimulation; SLK, superficial lamellar keratectomy; SPRR1a, small proline-rich repeat protein 1A; NGF, nerve growth factor; TENS, transcutaneous electrical nerve stimulation; ITNS, intranasal neurostimulation; DED, dry eye disease; EXNS, extranasal neurostimulation.

## Discussion

### Limitations and future directions

Electrotherapy is contraindicated in patients who are pregnant, epileptic, have cancer, or have cardiovascular disease. It is also contraindicated for those patients who have implanted electrical devices or have recently undergone radiotherapy due to unpredictable tissue response after radiotherapy [92]. Electrotherapy should also be avoided when skin is irritated, infected, bleeding or extremely sensitive [93]. This may narrow the number of patients who can be treated or participants for trials.

Currently, there are no standardized guidelines that exist for electrotherapy in NCP, causing a difference in the parameters used such as intensity, treatment duration and frequency. This makes the comparison of outcomes between studies much harder and hampers reproducibility. Insufficient stimulation may yield suboptimal outcomes, while excessive stimulation could result in adverse effects or diminishing returns. Determining the optimal dose of electrical therapy would be part of future research. Modification of ES according to the unique pathophysiology of corneal nerves may be necessary as it vastly differs from peripheral nerves.

In addition, the above-mentioned studies did not include a comparative arm, making it difficult to conclude whether the observed pain reduction was directly due to ES or attributed to spontaneous improvement or concurrent treatments. Future research should incorporate control groups to establish the cause-and-effect relationships concerning the efficacy of TENS.

Individual differences in the underlying etiology of NCP may also influence the effectiveness of electrical therapy. Factors such as baseline nerve damage, pain sensitivity, and response to treatment may lead to inconsistent outcomes. Moreover, most studies focus on short-term outcomes, with limited data on long-term efficacy and safety. It remains unclear whether the advantages of electrical therapy are sustained over time or require continuous treatment. The need for repeated sessions of electrotherapy over weeks or months may impose a logistical and financial burden on patients.

Furthermore, the use of electrotherapy requires specialized equipment and trained personnel, which may not be readily available in all healthcare settings, limiting its widespread adoption, particularly in low-resource settings and third-world countries. Reliance on subjective assessments, such as pain scores and patient-reported outcomes, may introduce bias. Objective measures, like corneal nerve imaging or electrophysiological data, are less frequently used, making it difficult to assess and adjust therapy dynamically.

Future directions can focus on investigating optimal parameters for various electrotherapy modalities such as TENS or trigeminal nerve stimulation. These can include intensity, duration, and frequency in respective patients to achieve maximal therapeutic outcomes. More effort should also be put in developing individualized electrotherapy regimens based on patient-specific factors so that it is catered to specific patient needs. The combined use of electrotherapy and pharmacological treatments should also be explored to maximize the efficacy of pain management.

Future studies should also aim to explore the molecular and cellular processes by which electrotherapy aids in pain alleviation and nerve regeneration. More detailed research into neurotrophic factors, such as BDNF and NGF, as well as calcium influx, could provide valuable insights. Moreover, follow-up studies can be performed to assess the lasting effects of electrotherapy in chronic NCP management and potential symptom recurrence. The safety of electrotherapy over extended periods while focusing on potential adverse effects can also be assessed on each patient, but objectively quantifying pain level and comfort might be hard. Long-term studies, larger-scale, and comparative or randomized controlled trials are required.

## Conclusion

Electrotherapy shows significant promise in the management of NCP. TENS has shown potential in reducing NCP severity, associated symptoms and improving corneal nerve function, indicating both symptomatic relief and recovery of nerves. The mechanism with which electrotherapy is used to treat peripheral neuropathic pain can be applied to NCP as well. Despite the promising results, challenges such as pain recurrence and the identification of optimal therapeutic parameters persist. The incorporation of electrotherapy into long-term management, especially for patients refractory to conventional treatments, warrants more research. While electrotherapy can emerge as a promising form of treatment in patients with NCP that offer benefits over current treatment, further research, including large-scale validation, clinical trials with control groups and longer follow-up period, and a deeper exploration of the molecular mechanisms involved, is critical to optimizing the safety and efficacy of electrotherapy in this clinical context.

## References

[1] Wong NSQ Liu C Lin MT Lee IXY Tong L Liu YC . Neuropathic corneal pain after coronavirus disease 2019 (COVID-19) infection. Diseases (2024) 12(2):37. 10.3390/diseases12020037 38391784 PMC10887979

[2] Muller LJ Marfurt CF Kruse F Tervo TM . Corneal nerves: structure, contents and function. Exp Eye Res (2003) 76(5):521–42. 10.1016/s0014-4835(03)00050-2 12697417

[3] Marfurt CF Cox J Deek S Dvorscak L . Anatomy of the human corneal innervation. Exp Eye Res (2010) 90(4):478–92. 10.1016/j.exer.2009.12.010 20036654

[4] Dieckmann G Goyal S Hamrah P . Neuropathic corneal pain: approaches for management. Ophthalmology (2017) 124(11):S34–S47. 10.1016/j.ophtha.2017.08.004 29055360 PMC5743225

[5] Belmonte C Nichols JJ Cox SM Brock JA Begley CG Bereiter DA TFOS DEWS II pain and sensation report. Ocul Surf (2017) 15(3):404–37. 10.1016/j.jtos.2017.05.002 28736339 PMC5706540

[6] Teo CHY Liu C Lee IXY Lin MTY Liu F Toh CJL Neuropathic corneal pain following refractive surgery: risk factors, clinical manifestations, imaging and proteomic characteristics. Br J Ophthalmol (2025) 109(7):747–55. 10.1136/bjo-2024-325996 39880672 PMC12229060

[7] Cheng J Liu C Yu M Lee IXY Wang X Hsu VWT Exploration of imaging and molecular biomarkers for differentiation of neuropathic corneal pain from dry eye syndrome. Ocul Surf (2025) 38:230–41. 10.1016/j.jtos.2025.08.002 40818751

[8] So WZ Wong NSQ Tan HC Yu Lin MT Yu Lee IX Mehta JS Diabetic corneal neuropathy as a surrogate marker for diabetic peripheral neuropathy. Neural Regen Res (2022) 17(10):2172–8. 10.4103/1673-5374.327364 35259825 PMC9083173

[9] Goyal S Hamrah P . Understanding neuropathic corneal Pain--Gaps and current therapeutic approaches. Semin Ophthalmol (2016) 31(1-2):59–70. 10.3109/08820538.2015.1114853 26959131 PMC5607443

[10] Chin JY Tong L Liu C Lee IXY Wong JHF Wong RKT Quality of life and symptomatology in neuropathic corneal pain in comparison with dry eye syndrome. Cornea. (2024) 44(7):825–31. 10.1097/ICO.0000000000003674 39160657 PMC12124199

[11] Liu C Lin MT Lee IXY Wong JHF Lu D Lam TC Neuropathic corneal pain: tear proteomic and neuromediator profiles, imaging features, and clinical manifestations. Am J Ophthalmol (2024) 265:6–20. 10.1016/j.ajo.2024.03.015 38521157

[12] Bron AJ de Paiva CS Chauhan SK Bonini S Gabison EE Jain S Tfos dews ii pathophysiology report. The Ocular Surface (2017) 15(3):438–510. 10.1016/j.jtos.2017.05.011 28736340

[13] Dieckmann G Ozmen MC Cox SM Engert RC Hamrah P . Low-dose naltrexone is effective and well-tolerated for modulating symptoms in patients with neuropathic corneal pain. The Ocular Surface (2021) 20:33–8. 10.1016/j.jtos.2020.12.003 33450415 PMC9009761

[14] Liu YC Lin MT Mehta JS . Analysis of corneal nerve plexus in corneal confocal microscopy images. Neural Regen Res (2021) 16(4):690–1. 10.4103/1673-5374.289435 33063728 PMC8067927

[15] Ross AR Al-Aqaba MA Almaazmi A Messina M Nubile M Mastropasqua L Clinical and *in vivo* confocal microscopic features of neuropathic corneal pain. Br J Ophthalmol (2020) 104(6):768–75. 10.1136/bjophthalmol-2019-314799 31533927

[16] Moein HR Akhlaq A Dieckmann G Abbouda A Pondelis N Salem Z Visualization of microneuromas by using *in vivo* confocal microscopy: an objective biomarker for the diagnosis of neuropathic corneal pain? Ocul Surf (2020) 18(4):651–6. 10.1016/j.jtos.2020.07.004 32663518 PMC7686058

[17] Guerrero-Moreno A Liang H Moreau N Luzu J Rabut G Melik Parsadaniantz S Corneal nerve abnormalities in painful dry eye disease patients. Biomedicines (2021) 9(10):1424. 10.3390/biomedicines9101424 34680542 PMC8533181

[18] Kocot-Kępska M Zajączkowska R Mika J Wordliczek J Dobrogowski J Przeklasa-Muszyńska A . Peripheral mechanisms of neuropathic pain—the role of neuronal and non-neuronal interactions and their implications for topical treatment of neuropathic pain. Pharmaceuticals (2021) 14(2):77. 10.3390/ph14020077 33498496 PMC7909513

[19] Van der Cruyssen F Politis C . Neurophysiological aspects of the trigeminal sensory system: an update. Rev Neurosciences (2018) 29(2):115–23. 10.1515/revneuro-2017-0044 29116936

[20] Opree A Kress M . Involvement of the proinflammatory cytokines tumor necrosis factor-alpha, IL-1 beta, and IL-6 but not IL-8 in the development of heat hyperalgesia: effects on heat-evoked calcitonin gene-related peptide release from rat skin. J Neurosci (2000) 20(16):6289–93. 10.1523/JNEUROSCI.20-16-06289.2000 10934280 PMC6772609

[21] Puja G Sonkodi B Bardoni R . Mechanisms of peripheral and central pain sensitization: focus on ocular pain. Front Pharmacol (2021) 12:764396. 10.3389/fphar.2021.764396 34916942 PMC8669969

[22] Yin Y Yi MH Kim DW . Impaired autophagy of GABAergic interneurons in neuropathic pain. Pain Res Manag (2018) 2018:9185368. 10.1155/2018/9185368 30356379 PMC6176324

[23] Castro A Li Y Raver C Chandra R Masri R Lobo MK Neuropathic pain after chronic nerve constriction may not correlate with chloride dysregulation in mouse trigeminal nucleus caudalis neurons. Pain (2017) 158(7):1366–72. 10.1097/j.pain.0000000000000926 28426550 PMC5482239

[24] Rahman M Okamoto K Thompson R Katagiri A Bereiter DA . Sensitization of trigeminal brainstem pathways in a model for tear deficient dry eye. Pain (2015) 156(5):942–50. 10.1097/j.pain.0000000000000135 25734990 PMC4402282

[25] Mills EP Alshelh Z Kosanovic D Di Pietro F Vickers ER Macey PM Altered brainstem pain-modulation circuitry connectivity during spontaneous pain intensity fluctuations. J Pain Res (2020) 13:2223–35. 10.2147/JPR.S252594 32943915 PMC7481287

[26] Huang JJ Rodriguez DA Slifer SH Martin ER Levitt RC Galor A . Genome wide association study of neuropathic ocular pain. Ophthalmol Sci (2024) 4(2):100384. 10.1016/j.xops.2023.100384 37868788 PMC10587615

[27] Belin S Maki BA Catlin J Rein BA Popescu GK . Membrane stretch gates NMDA receptors. J Neurosci (2022) 42(29):5672–80. 10.1523/JNEUROSCI.0350-22.2022 35705487 PMC9302457

[28] Woo S-H Lukacs V De Nooij JC Zaytseva D Criddle CR Francisco A Piezo2 is the principal mechanotransduction channel for proprioception. Nat Neuroscience (2015) 18(12):1756–62. 10.1038/nn.4162 26551544 PMC4661126

[29] Eijkelkamp N Linley JE Torres JM Bee L Dickenson AH Gringhuis M A role for Piezo2 in EPAC1-dependent mechanical allodynia. Nat Commun (2013) 4:1682. 10.1038/ncomms2673 23575686 PMC3644070

[30] Zhang M Wang Y Geng J Zhou S Xiao B . Mechanically activated piezo channels mediate touch and suppress acute mechanical pain response in mice. Cell Rep (2019) 26(6):1419–31 e4. 10.1016/j.celrep.2019.01.056 30726728

[31] Sonkodi B Resch MD Hortobágyi T . Is the sex difference a clue to the pathomechanism of dry eye disease? Watch out for the NGF-TrkA-Piezo2 signaling axis and the Piezo2 channelopathy. J Mol Neurosci (2022) 72(8):1598–608. 10.1007/s12031-022-02015-9 35507012 PMC9374789

[32] Puja G Sonkodi B Bardoni R . Mechanisms of peripheral and central pain sensitization: focus on ocular. Eye Pain: Etiology Ther Approaches (2022):78852468. 10.3389/fphar.2021.764396 PMC8669969 34916942

[33] Fernández-Trillo J Florez-Paz D Íñigo-Portugués A González-González O Del Campo AG González A Piezo2 mediates low-threshold mechanically evoked pain in the cornea. J Neurosci (2020) 40(47):8976–93. 10.1523/JNEUROSCI.0247-20.2020 33055278 PMC7673006

[34] Matynia A Nguyen E Sun X Blixt FW Parikh S Kessler J Peripheral sensory neurons expressing melanopsin respond to light. Front Neural Circuits (2016) 10:60. 10.3389/fncir.2016.00060 27559310 PMC4978714

[35] Asiedu K . Role of ocular surface neurobiology in neuronal-mediated inflammation in dry eye disease. Neuropeptides (2022) 95:102266. 10.1016/j.npep.2022.102266 35728484

[36] Guerrero-Moreno A Baudouin C Melik Parsadaniantz S Réaux-Le Goazigo A . Morphological and functional changes of corneal nerves and their contribution to peripheral and central sensory abnormalities. Front Cellular Neuroscience (2020) 14:610342. 10.3389/fncel.2020.610342 33362474 PMC7758484

[37] Blanco-Vázquez M Vázquez A Fernández I Novo-Diez A Martínez-Plaza E García-Vázquez C Inflammation-related molecules in tears of patients with chronic ocular pain and dry eye disease. Exp Eye Res (2022) 219:109057. 10.1016/j.exer.2022.109057 35358536

[38] Li B Tian Y Wang S . The correlation of cytokines and sensory hypersensitivity in mild dry eye patients characterized by symptoms outweighing signs. Mol Vision (2020) 26:359–69. 32476816 PMC7245605

[39] Launay P-S Reboussin E Liang H Kessal K Godefroy D Rostene W Ocular inflammation induces trigeminal pain, peripheral and central neuroinflammatory mechanisms. Neurobiol Disease (2016) 88:16–28. 10.1016/j.nbd.2015.12.017 26747211

[40] Ren K Dubner R . Interactions between the immune and nervous systems in pain. Nat Medicine (2010) 16(11):1267–76. 10.1038/nm.2234 20948535 PMC3077564

[41] Asiedu K . Neurophysiology of corneal neuropathic pain and emerging pharmacotherapeutics. J Neurosci Res (2024) 102(1):e25285. 10.1002/jnr.25285 38284865

[42] Khan N Smith MT . Neurotrophins and neuropathic pain: role in pathobiology. Molecules (2015) 20(6):10657–88. 10.3390/molecules200610657 26065639 PMC6272404

[43] Yang LWY Mehta JS Liu YC . Corneal neuromediator profiles following laser refractive surgery. Neural Regen Res (2021) 16(11):2177–83. 10.4103/1673-5374.308666 33818490 PMC8354117

[44] de Paiva CS Pflugfelder SC . Rationale for anti-inflammatory therapy in dry eye syndrome. Arq Bras Oftalmol. (2008) 71(6 Suppl. l):89–95. 10.1590/s0004-27492008000700017 19274418

[45] Anam A Liu C Tong L Liu YC . Blood-derived eye drops for the treatment of corneal neuropathic pain. J Ocul Pharmacol Ther (2024) 40(5):281–92. 10.1089/jop.2023.0155 38648544 PMC11296151

[46] Dworkin RH O'Connor AB Kent J Mackey SC Raja SN Stacey BR Interventional management of neuropathic pain: NeuPSIG recommendations. Pain (2013) 154(11):2249–61. 10.1016/j.pain.2013.06.004 23748119 PMC4484720

[47] Dimit R Gire A Pflugfelder SC Bergmanson JP . Patient ocular conditions and clinical outcomes using a PROSE scleral device. Cont Lens Anterior Eye (2013) 36(4):159–63. 10.1016/j.clae.2013.02.004 23499361

[48] Moisset X Lanteri-Minet M Fontaine D . Neurostimulation methods in the treatment of chronic pain. J Neural Transm (Vienna) (2020) 127(4):673–86. 10.1007/s00702-019-02092-y 31637517

[49] Chu X-L Song X-Z Li Q Li YR He F Gu XS Basic mechanisms of peripheral nerve injury and treatment *via* electrical stimulation. Neural Regeneration Research (2022) 17(10):2185–93. 10.4103/1673-5374.335823 35259827 PMC9083151

[50] Sabino GS Santos CM Francischi JN De Resende MA . Release of endogenous opioids following transcutaneous electric nerve stimulation in an experimental model of acute inflammatory pain. The Journal Pain (2008) 9(2):157–63. 10.1016/j.jpain.2007.09.003 17988952

[51] Sluka KA Lisi TL Westlund KN . Increased release of serotonin in the spinal cord during low, but not high, frequency transcutaneous electric nerve stimulation in rats with joint inflammation. Arch Physical Medicine Rehabilitation (2006) 87(8):1137–40. 10.1016/j.apmr.2006.04.023 16876561 PMC2746636

[52] Maeda Y Lisi T Vance C Sluka K . Release of GABA and activation of GABAA in the spinal cord mediates the effects of TENS in rats. Brain Research (2007) 1136:43–50. 10.1016/j.brainres.2006.11.061 17234163 PMC2746639

[53] Song Y Guo Y Zhao L Chen S . Electroacupuncture alleviates inflammatory pain via adenosine suppression and its mediated substance P expression. Arquivos de Neuro-Psiquiatria (2020) 78:617–23. 10.1590/0004-282X20200078 33146290

[54] Melzack R Wall PD . Pain mechanisms: a new theory. Science (1965) 150(3699):971–9. 10.1126/science.150.3699.971 5320816

[55] Vance CG Dailey DL Rakel BA Sluka KA . Using TENS for pain control: the state of the evidence. Pain Manag (2014) 4(3):197–209. 10.2217/pmt.14.13 24953072 PMC4186747

[56] Johnson M . Transcutaneous electrical nerve stimulation: mechanisms, clinical application and evidence. Rev Pain (2007) 1(1):7–11. 10.1177/204946370700100103 26526976 PMC4589923

[57] Billington R . Consequences of neuropathic pain: quality-of-life issues and associated costs. Am J Manag Care (2006) 12(9 Suppl. l):S263–8. 16774458

[58] Sluka KA Walsh D . Transcutaneous electrical nerve stimulation: basic science mechanisms and clinical effectiveness. J Pain (2003) 4(3):109–21. 10.1054/jpai.2003.434 14622708

[59] Chen CC Johnson MI McDonough S Cramp F . The effect of transcutaneous electrical nerve stimulation on local and distal cutaneous blood flow following a prolonged heat stimulus in healthy subjects. Clin Physiology Functional Imaging (2007) 27(3):154–61. 10.1111/j.1475-097X.2007.00731.x 17445066

[60] Somers DL Somers MF . Treatment of neuropathic pain in a patient with diabetic neuropathy using transcutaneous electrical nerve stimulation applied to the skin of the lumbar region. Phys Therapy (1999) 79(8):767–75. 10440663

[61] Forst T Nguyen M Forst S Disselhoff B Pohlmann T Pfützner A . Impact of low frequency transcutaneous electrical nerve stimulation on symptomatic diabetic neuropathy using the new salutaris device. Diabetes Nutrition and Metabolism (2004) 17(3):163–8. 15334794

[62] Zhao M Bai H Wang E Forrester JV McCaig CD . Electrical stimulation directly induces pre-angiogenic responses in vascular endothelial cells by signaling through VEGF receptors. J Cell Science (2004) 117(3):397–405. 10.1242/jcs.00868 14679307 PMC1459284

[63] Hughes JGS Lichstein PR Whitlock D Harker C . Response of plasma beta-endorphins to transcutaneous electrical nerve stimulation in healthy subjects. Phys Therapy (1984) 64(7):1062–6. 10.1093/ptj/64.7.1062 6330773

[64] Ding L Song T Yi C Huang Y Yu W Ling L Transcutaneous electrical nerve stimulation (TENS) improves the diabetic cytopathy (DCP) via up-regulation of CGRP and cAMP. PLoS One (2013) 8(2):e57477. 10.1371/journal.pone.0057477 23468996 PMC3585412

[65] Zhou W-t Ni Y-q Jin Z-b Zhang M Wu Jh. Zhu Y Electrical stimulation ameliorates light-induced photoreceptor degeneration *in vitro via* suppressing the proinflammatory effect of microglia and enhancing the neurotrophic potential of müller cells. Exp Neurology (2012) 238(2):192–208. 10.1016/j.expneurol.2012.08.029 22974557

[66] ElZaridi F Lennikov A Cho K-S Dartt DA Yang M Chen D . Electrical stimulation promotes growth of the corneal nerves. Invest Ophthalmol and Vis Sci (2023) 64(8):5402.

[67] DeSantana JM Walsh DM Vance C Rakel BA Sluka KA . Effectiveness of transcutaneous electrical nerve stimulation for treatment of hyperalgesia and pain. Curr Rheumatol Rep (2008) 10(6):492–9. 10.1007/s11926-008-0080-z 19007541 PMC2746624

[68] Kumar D Marshall HJ . Diabetic peripheral neuropathy: amelioration of pain with transcutaneous electrostimulation. Diabetes Care (1997) 20(11):1702–5. 10.2337/diacare.20.11.1702 9353612

[69] Gibson W Wand BM O'Connell NE . Transcutaneous electrical nerve stimulation (TENS) for neuropathic pain in adults. Cochrane Database Syst Rev (2017)(9). 10.1002/14651858.CD011976.pub2 PMC6426434 28905362

[70] Ni L Yao Z Zhao Y Zhang T Wang J Li S Electrical stimulation therapy for peripheral nerve injury. Front Neurol (2023) 14:1081458. 10.3389/fneur.2023.1081458 36908597 PMC9998520

[71] Brooks J Tracey I . From nociception to pain perception: imaging the spinal and supraspinal pathways. J Anat (2005) 207(1):19–33. 10.1111/j.1469-7580.2005.00428.x 16011543 PMC1571498

[72] Sayegh RR Sweet JA Miller JP Hayek SM . Electrical stimulation of the trigeminal ganglion and intrathecal drug delivery systems for the management of corneal neuropathic pain. Cornea (2016) 35(4):576–7. 10.1097/ICO.0000000000000751 26807903

[73] Yoo Y-S Park S Eun P Park YM Lim DH Chung T-Y . Corneal neuro-regenerative effect of transcutaneous electrical stimulation in rabbit lamellar keratectomy model. Translational Vis Sci and Technology (2022) 11(10):17. 10.1167/tvst.11.10.17 36223127 PMC9583744

[74] Ghaffariyeh A Peyman A Puyan S Honarpisheh N Bagheri B Peyman M . Evaluation of transcutaneous electrical simulation to improve recovery from corneal hypoesthesia after LASIK. Graefe's Archive Clin Exp Ophthalmol (2009) 247(8):1133–8. 10.1007/s00417-009-1079-5 19468742

[75] Yan X Liu J Huang J Huang M He F Ye Z Electrical stimulation induces calcium-dependent neurite outgrowth and immediate early genes expressions of dorsal root ganglion neurons. Neurochem Res (2014) 39(1):129–41. 10.1007/s11064-013-1197-7 24248860

[76] Liu Y Fox PM . The role of electrical stimulation in peripheral nerve regeneration: current evidence and future directions. J Hand Surg Glob Online (2024) 6(5):718–21. 10.1016/j.jhsg.2024.01.022 39381378 PMC11456620

[77] Zayan K Aggarwal S Felix E Levitt R Sarantopoulos K Galor A . Transcutaneous electrical nerve stimulation for the long-term treatment of ocular pain. Neuromodulation (2020) 23(6):871–7. 10.1111/ner.13146 32196838 PMC7483841

[78] Liao CD Tsauo JY Liou TH Chen HC Rau CL . Efficacy of noninvasive stellate ganglion blockade performed using physical agent modalities in patients with sympathetic hyperactivity-associated disorders: a systematic review and meta-analysis. PLoS One (2016) 11(12):e0167476. 10.1371/journal.pone.0167476 27911934 PMC5135105

[79] Cohn GS Corbett D Tenen A Coroneo M McAlister J Craig JP Randomized, controlled, double-masked, multicenter, pilot study evaluating safety and efficacy of intranasal neurostimulation for dry eye disease. Invest Ophthalmol and Vis Sci (2019) 60(1):147–53. 10.1167/iovs.18-23984 30629728

[80] Brinton M Kossler AL Patel ZM Loudin J Franke M Ta CN Enhanced tearing by electrical stimulation of the anterior ethmoid nerve. Invest Ophthalmol and Vis Sci (2017) 58(4):2341–8. 10.1167/iovs.16-21362 28431436 PMC5398789

[81] Gumus K Schuetzle KL Pflugfelder SC . Randomized controlled crossover trial comparing the impact of sham or intranasal tear neurostimulation on conjunctival goblet cell degranulation. Am Journal Ophthalmology (2017) 177:159–68. 10.1016/j.ajo.2017.03.002 28302532 PMC5457166

[82] Farhangi M Cheng AM Baksh B Sarantopoulos CD Felix ER Levitt RC Effect of non-invasive intranasal neurostimulation on tear volume, dryness and ocular pain. Br J Ophthalmol (2020) 104(9):1310–6. 10.1136/bjophthalmol-2019-315065 31831506

[83] Mendell LM . Constructing and deconstructing the gate theory of pain. Pain® (2014) 155(2):210–6. 10.1016/j.pain.2013.12.010 24334188 PMC4009371

[84] Olcucu O Dieckmann G Ozmen MC Cox S Liu G Hamrah P . An exploratory, prospective, interventional, open-label, clinical trial with intranasal neurostimulation for ameliorating symptoms of neuropathic corneal pain. Invest Ophthalmol and Vis Sci (2024) 65(7):2658.

[85] Ji MH Moshfeghi DM Periman L Kading D Matossian C Walman G Novel extranasal tear stimulation: pivotal study results. Translational Vision Science and Technology (2020) 9(12):23. 10.1167/tvst.9.12.23 33244443 PMC7683850

[86] Moayedi M Davis KD . Theories of pain: from specificity to gate control. J Neurophysiology (2013) 109(1):5–12. 10.1152/jn.00457.2012 23034364

[87] Koseoglu ND Chen E Tuwani R Kompa B Cox SM Cuneyt Ozmen M Development and validation of a deep learning model for diagnosing neuropathic corneal pain *via in vivo* confocal microscopy. Npj Digital Med (2025) 8(1):277. 10.1038/s41746-025-01577-3 40369269 PMC12078480

[88] Smith TJ Wang EJ Loprinzi CL . Cutaneous electroanalgesia for relief of chronic and neuropathic pain. New Engl J Med (2023) 389(2):158–64. 10.1056/NEJMra2110098 37437145

[89] Lee SY Park C-H Cho YS Kim L Yoo JW Joo SY Scrambler therapy for chronic pain after burns and its effect on the cerebral pain network: a prospective, double-blinded, randomized controlled trial. J Clin Med (2022) 11(15):4255. 10.3390/jcm11154255 35893347 PMC9332864

[90] Starkweather AR Coyne P Lyon DE Elswick JR An K Sturgill J . Decreased low back pain intensity and differential gene expression following calmare®: results from a double‐blinded randomized sham‐controlled study. Res Nursing and Health (2015) 38(1):29–38. 10.1002/nur.21632 25572279

[91] Karakus S Rajabalee N Tunc U Smith TJ . Scrambler therapy as a novel treatment for unilateral ocular neuropathic pain. The Ocular Surface (2024) 34:122–3. 10.1016/j.jtos.2024.06.006 38950774

[92] Radhakrishnan R Sluka KA . Spinal muscarinic receptors are activated during low or high frequency TENS-induced antihyperalgesia in rats. Neuropharmacology (2003) 45(8):1111–9. 10.1016/s0028-3908(03)00280-6 14614954 PMC2746650

[93] Almalty AR Hamed SH Jebril MY Abdelnour HM . The effect of electrical stimulation on skin vulnerability to irritants. Skin Res Technol (2024) 30(2):e13591. 10.1111/srt.13591 38279544 PMC10818122

